# A *Poisson *mixture model to identify changes in RNA polymerase II binding quantity using high-throughput sequencing technology

**DOI:** 10.1186/1471-2164-9-S2-S23

**Published:** 2008-09-16

**Authors:** Weixing Feng, Yunlong Liu, Jiejun Wu, Kenneth P Nephew, Tim HM Huang, Lang Li

**Affiliations:** 1 Division of Biostatistics, Indiana University School of Medicine, Indianapolis, IN 46202, USA; 2 Center for Computational Biology and Bioinformatics, Indiana University School of Medicine, Indianapolis, IN 46202, USA; 3 Center for Medical Genomics, Indiana University School of Medicine, Indianapolis, IN 46202, USA; 4 College of Automation, Harbin Engineering University, Harbin, Heilongjiang 150001 PR China; 5 Medical Sciences, Indiana University School of Medicine, Bloomington, IN 47405, USA; 6 Departments of Cellular and Integrative Physiology, Indiana University School of Medicine, Indianapolis, IN 46202, USA; 7 IU Simon Cancer Center, Indianapolis, IN 46202, USA; 8 Division of Human Cancer Genetics, Department of Molecular Virology, Immunology, and Medical Genetics, Comprehensive Cancer Center, Ohio State University, Columbus, OH 43210, USA

## Abstract

We present a mixture model-based analysis for identifying differences in the distribution of RNA polymerase II (Pol II) in transcribed regions, measured using ChIP-seq (chromatin immunoprecipitation following massively parallel sequencing technology). The statistical model assumes that the number of Pol II-targeted sequences contained within each genomic region follows a *Poisson *distribution. A *Poisson *mixture model was then developed to distinguish Pol II binding changes in transcribed region using an empirical approach and an expectation-maximization (EM) algorithm developed for estimation and inference. In order to achieve a global maximum in the M-step, a particle swarm optimization (PSO) was implemented. We applied this model to Pol II binding data generated from hormone-dependent MCF7 breast cancer cells and antiestrogen-resistant MCF7 breast cancer cells before and after treatment with 17*β*-estradiol (E2). We determined that in the hormone-dependent cells, ~9.9% (2527) genes showed significant changes in Pol II binding after E2 treatment. However, only ~0.7% (172) genes displayed significant Pol II binding changes in E2-treated antiestrogen-resistant cells. These results show that a *Poisson *mixture model can be used to analyze ChIP-seq data.

## Introduction

Massively parallel sequencing is a high-throughput technology capable of sequencing hundreds of thousands of DNA fragments in a single experiment. Combined with antibody-based chromatin immunoprecipitation assay (or ChIP-seq assay), this technology has been demonstrated to be a comprehensive, quantitative and cost-effective approach for mapping protein-DNA interaction on a genome-wide scale [1]. One ChIP-seq run can generate more than 20 million sequence tags of up to 36 bps each, which can then be definitively mapped to the human genome.

Due to the large amount of data generated in high-throughput sequencing experiments, innovative computational and statistical approaches are required to identify biological signals from ChIP-seq data. To date, several approaches have been applied to identify genomic regions containing a high concentration of sequence hits, i.e., "ChIP-seq peaks" [1]. An underlying assumption of current approaches is that DNA-binding proteins, such as transcription factors, contain sequence-specific, DNA binding domains that target a cluster of *cis*-acting DNA elements sharing certain sequence features. While such algorithms can identify DNA binding sites for highly specific transcription factors, current approaches are not appropriate for identifying binding sites for the general transcriptional machinery, such as RNA polymerase II (Pol II), which typically does not display high sequence specificity. In addition, as Pol II activity likely extends beyond the promoter/transcription start site of active genes, algorithms for assessing long-range Pol II binding are needed. Therefore, in this study, we have proposed a mixture model-based analysis for identifying differences in Pol II distribution in combined 5'-end, open reading frame (ORF), and 3'-untranscribed regions of active genes. Our strategy is based on the underlying assumption that the number of Pol II-target sequences follows a *Poisson *distribution and can be used to identify differentially transcribed genes under different experimental conditions. Furthermore, our proposed methodology can be used for making statistical inferences in experiments for which replicates are not available.

To date statistical models for ChIP-seq data are very limited. Nevertheless, new algorithms for ChIP-seq can be developed using existing framework for identifying differentially expressed genes from microarray data. For example, Kerr *et al*. [2] and Wolfinger *et al*. [3] employed analysis of variance (ANOVA) models to conduct a hypothetical test for different expression levels of individual genes in multiple microarray experiments. Dudoit *et al*. [4] used a *t-*statistic to address the problem of multiple comparisons through permutation analysis. These approaches yielded only *p*-values representing the probability of having an observed expression difference of a given gene, if the status is assumed to be the same before and after a treatment. However, for Pol II binding data derived from ChIP-seq, these approaches cannot be used to estimate the status change. A more appropriate choice analyzing ChIP-seq Pol II binding data may be a *Bayes *or an empirical *Bayes *approach. In this regard, Efron *et al*. [5] proposed an empirical *Bayes *model for calculating the probability of a differentially expressed gene given the observed data. As the empirical distribution for their t-like statistic for each gene does not share variation information, it works well only in situations involving at least a few replicates. Newton *et al*. [6] proposed another empirical *Bayes *model for cDNA microarray experiments with only one replicate. According to their method, if a gene is differentially expressed under two conditions, its level of expression is independently generated from the same distribution [6]; otherwise, the level of expression is the same between experimental and control samples. In their work, the observational component follows a *Gamma *distribution with mean *μ*_*g*_, and *μ*_*g *_itself follows an inverse *Gamma *distribution (prior component). This hierarchical model is often referred to as the *Gamma-Gamma *model. Specifically, all genes share the same distribution for the within-gene sampling errors, a crucial feature in their method, as no replicates were available in their data example. Kendziorski *et al*. [7] further extended the *Gamma-Gamma *model to situations where replicates were available. In addition, they developed a log-normal model for the observational component and a normal model for the prior component. They demonstrated a comparable performance for a lognormal-normal model and a *Gamma-Gamma *model. A major advantage in the methods proposed by Newton *et al. *[6] and Kendziorski *et al*. [7] is that information sharing is a consequence of the empirical *Bayes *approach. The model pools the variation information across all genes, making it well suited for data sets containing only a few replicates (e.g., 2 replicates), and we have successfully utilized this model framework to test the correlation among genome wide gene expression, DNA methylation, and histone acetylation [8,9].

In the current paper, we propose a different model, *Poisson *mixture model, within the same empirical *Bayes *framework for identifying gene targets with differential Pol II binding activities in breast cancer MCF7 cell line under various conditions. ChIP-seq data processing, normalization, and statistical methods are proposed in the method section; analysis of ChIP-seq data from breast cancer cell lines (MCF7 and its tamoxifen-resistant subline OHT-MCF7) before and after treatment with 17*β*-estradial (E2), are presented in the result section, with conclusions at the end of the paper.

## Methods

As described by Fan et al [10], MCF7 human breast cancer cells (American Type Culture Collection, Manassas, VA) and MCF7-OTH cells were cultured and treated with E2 (10^-8 ^mol/L) for three hours. Then, cells were cross-linked with 1% formaldehyde and chromatin immunoprecipitation was done as previously described [11]. The antibodies against Pol II were purchased from Santa Cruz Biotechnology (Santa Cruz, sc-899 X and sc-8005 X). After immunoprecipitation and purification, ChIP DNA sample was run in 12% PAGE and the 100–300 bp DNA fraction was excised and eluted from the gel slice. Then, Illumina library was constructed and sequenced with Illumina/Solexa Genome Analyzer.

**Figure 1:**
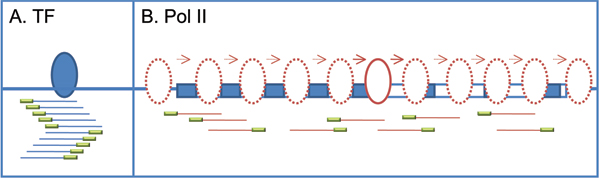
Schematics of ChIP-seq-derived DNA fragments targeting transcription factors and RNA polymerase II. Blue and red ellipses indicate transcription factors binding on specific *cis*-acting DNA element, and RNA polymerase II not targeting certain binding sites, respectively. Blue and red lines under the ellipses illustrate sheared DNA fragments bound by the DNA-binding protein and pulled down by the immunoprecipitation assay. Green box indicates the fragment derived by Solexa sequencing (~25–36 bp).

Most DNA binding proteins, such as transcription factors, bind to *cis*-acting DNA element with specific sequence features usually described by a position weight matrix (PWM). A hypothetical distribution of ChIP-seq-derived DNA fragments corresponding to transcription factors and RNA polymerase II (Pol II) is shown in Figs. 1A and 1B, respectively. For transcription factors, ChIP-seq detects a set of fragments that cluster and center around distinct biological binding sites, forming a "peak" around the binding locus (Fig. 1A). In contrast, Pol II binds throughout promoter regions, the 5'- and 3'- untranslated regions, the open reading frame (ORF), and downstream regions of the activated gene (Fig. 1B). Although Pol II can form a distinct peak around the transcription start site under certain circumstances, commonly-used peak-finding algorithms are not able to identify Pol II-enriched regions in gene transcript region derived from ChIP-seq experiments.

### A *Poisson *mixture model to identify transcripts with different Pol II binding quantity

Based on the assumption that the number of Pol II-binding fragments detected within gene transcript region, including 5'- and 3'-untranslated regions and open reading frames, follows a *Poisson *distribution, we developed a mixture model to identify differences in Pol II binding under two conditions, control *vs. *treatment. Denote *y*_*ij *_as the Pol II quantity for gene *i *(i = 1,..., n) under condition *j *(*j *= 1, 2). In this application, *j *= 1, 2 indicates two biological conditions, MCF7 control (vehicle-treated) and MCF7 treated (3 hour E2 treatment), respectively. Marginally, *y*_*ij *_follows a *Poisson *distribution,

(1) $$y_{ij}\sim \frac{e^{-\lambda_{ij}}\lambda_{ij}^{y_{ij}}}{y_{ij}!},\lambda_{ij}\geq 0$$

where *λ*_*ij *_denotes the expected quantity of Pol II binding for gene *i *under condition *j *(*j *= 1, 2). For the *i*-th gene, if Pol II binding quantities within the gene transcript region are statistically different between the two biological conditions, *y*_*ij *_follows marginal distributions with different parameters *λ*_*i*1 _and *λ*_*i*2_; conversely, if the number of Pol II binding does not demonstrate a significant difference, *y*_*ij *_follows the same distribution with a unified *λ*. A mixture model is proposed to estimate the posterior probability of differential Pol II binding quantity.

For unified Pol II binding quantity between two conditions, *λ*_*i*1 _and *λ*_*i*2 _follow the same *Gamma *distribution (2). The selection of *Gamma *distribution is based on two considerations. Firstly, the two parameter *Gamma *distribution is a very flexible function which can be used to describe a wide range of distribution shapes. Secondly, the *Gamma *distribution is a conjugate distribution of *Poisson *for the *λ *parameters. Hence, the follow-up expectation step in an E-M algorithm has a close form solution.

(2) $$\lambda_{i1}=\lambda_{i2}=\lambda_{i}\sim Gamma(\alpha_{1},\beta_{1})=\frac{\lambda_{i}^{\alpha_{1}-1}e^{-\lambda_{i}/\beta_{1}}}{\Gamma (\alpha_{1})\beta_{1}^{\alpha_{1}}}.$$

Otherwise, (*λ*_*i*1_, *λ*_*i*2_) are distributed independently.

(3) $$\begin{array}{c} \lambda_{i1}\sim Gamma(\alpha_{1},\beta_{1})=\frac{\lambda_{i1}^{\alpha_{1}-1}e^{-\lambda_{i1}/\beta_{1}}}{\Gamma (\alpha_{1})\beta_{1}^{\alpha_{1}}}\\ \lambda_{i2}\sim Gamma(\alpha_{2},\beta_{2})=\frac{\lambda_{i2}^{\alpha_{2}-1}e^{-\lambda_{i2}/\beta_{2}}}{\Gamma (\alpha_{2})\beta_{2}^{\alpha_{2}}} \end{array}$$

Denote *Z*_*i *_as the *Bernoulli *random variable with probability *p*, *i.e*., it is equal to 1 if (*λ*_*i*1_, *λ*_*i*2_) are independently distributed (and equal to 0 if not). Therefore, the joint distribution of Pol II binding quantity is modeled by a mixture of uniform binding events (*p *= 0) and differential binding events (*p *= 1).

(4) $$\begin{array}{l} \mathrm{Pr}(y,\lambda ,Z|\alpha_{1},\beta_{1},\alpha_{2},\beta_{2},P)\\ =\prod_{i=1}^{n}\mathrm{Pr}(y_{i1},y_{i2},\lambda_{i},\lambda_{i1},\lambda_{i2}|Z_{i},\alpha_{1},\beta_{1},\alpha_{2},\beta_{2})\mathrm{Pr}(Z_{i}|p)\\ =\prod_{i=1}^{n}{\left(1-P\right)}^{\left(1-Z_{i}\right)}{\left[\frac{e^{-\lambda_{i}}\lambda_{i}^{y_{i1}+y_{i2}}e^{-\lambda_{i}/\beta_{1}}\lambda_{i}^{\alpha_{1}-1}}{y_{i1}!y_{i2}!\Gamma (\alpha_{1})\beta_{1}^{\alpha_{1}}}\right]}^{1-Z_{i}}\times \\ P^{Z_{i}}\left[\frac{e^{-2\lambda_{i1}}\lambda_{i1}^{y_{i1}}e^{-\lambda_{i1}/\beta_{1}}\lambda_{i1}^{\alpha_{1}-1}}{y_{i1}!\Gamma (\alpha_{1})\beta_{1}^{\alpha_{1}}}\times \frac{e^{-\lambda_{i2}}\lambda_{i2}^{y_{i2}}e^{-\lambda_{i2}/\beta_{2}}\lambda_{i2}^{\alpha_{2}-1}}{y_{i2}!\Gamma (\alpha_{2})\beta_{2}^{\alpha_{2}}}\right] \end{array}$$

Based on equation (4), we implement E-M algorithm by treating *Z*_*i *_as missing data.

The **E-step **of the algorithm is specified as:

(5) $$\begin{array}{l} {\hat{\lambda }}_{i}=E(\lambda_{i}|Z_{i})=(y_{i1}+y_{i2}+\alpha_{1})\times (1/(2+1/\beta_{1}))\\ {\hat{\lambda }}_{i1}=E(\lambda_{i1}|Z_{i})=(y_{i1}+\alpha_{1})\times (1/(1+1/\beta_{1}))\\ {\hat{\lambda }}_{i2}=E(\lambda_{i2}|Z_{i})=(y_{i2}+\alpha_{2})\times (1/(1+1/\beta_{2}))\\ {\hat{Z}}_{i}=\frac{p\left[\frac{e^{-(1+1/\beta_{1}){\hat{\lambda }}_{i1}}{\hat{\lambda }}_{i1}^{y_{i1}+\alpha_{1}-1}e^{-(1+1/\beta_{2}){\hat{\lambda }}_{i2}}{\hat{\lambda }}_{i2}^{y_{i2}+\alpha_{2}-1}}{y_{i1}!\Gamma (\alpha_{1})\beta_{1}^{\alpha_{1}}y_{i2}!\Gamma (\alpha_{2})\beta_{2}^{\alpha_{2}}}\right]}{p\left[\frac{e^{-(1+1/\beta_{1}){\hat{\lambda }}_{i1}}{\hat{\lambda }}_{i1}^{y_{i1}+\alpha_{1}-1}e^{-(1+1/\beta_{2}){\hat{\lambda }}_{i2}}{\hat{\lambda }}_{i2}^{y_{i2}+\alpha_{2}-1}}{y_{i1}!\Gamma (\alpha_{1})\beta_{1}^{\alpha_{1}}y_{i2}!\Gamma (\alpha_{2})\beta_{2}^{\alpha_{2}}}\right]+(1-p)\frac{e^{-(2+1/\beta_{1}){\hat{\lambda }}_{i}}{\hat{\lambda }}_{i}^{y_{i1}+y_{i2}+\alpha_{1}-1}}{y_{i1}!y_{i2}!\Gamma (\alpha_{1})\beta_{1}^{\alpha_{1}}}} \end{array}$$

In the **M-step**, the parameters in the *Gamma *distribution (*α*_1_, *β*_1_, *α*_2_, *β*_2_) are estimated by maximizing the two likelihood functions in Equation 6.

(6) $$\begin{array}{l} L(\alpha_{1},\beta_{1};\lambda ,Z)\propto \prod_{i=1}^{n}\left\{{\left[\frac{e^{-\lambda_{i}/\beta_{1}}\lambda_{i}^{\alpha_{1}-1}}{\Gamma (\alpha_{1})\beta_{1}^{\alpha_{1}}}\right]}^{1-Z_{i}}{\left[\frac{e^{-\lambda_{i1}/\beta_{1}}\lambda_{i1}^{\alpha_{1}-1}}{\Gamma (\alpha_{1})\beta_{1}^{\alpha_{1}}}\right]}^{Z_{i}}\right\}\\ L(\alpha_{2},\beta_{2};\lambda ,Z)\propto \prod_{i=1}^{n}\left\{{\left[\frac{e^{-\lambda_{i2}/\beta_{2}}\lambda_{i2}^{\alpha_{2}-1}}{\Gamma (\alpha_{2})\beta_{2}^{\alpha_{2}}}\right]}^{Z_{i}}\right\}\\ p=\frac{\sum_{i=1,2,...,n}Z_{i}}{n} \end{array}$$

In the M-step, the optimization procedure is challenging, because searching for the optimal solutions for *Gamma *parameters can be trapped into local optimum, causing either slow convergence or failure to converge on the global optimal solution. In order to overcome these difficulties, we utilize particle swarm optimization (PSO), an artificial intelligence approach that mimics a behavior of swarm-forming agents, providing a good balance between global optimum searching and computation efficiency [12].

Because the likelihood functions of (*α*_1_, *β*_1_) and (*α*_2_, *β*_2_) are factorized in equation 6, the PSO optimization procedures are conducted independently using the following four steps.

Step 1: 100 particles (potential solution) were initially randomly distributed in 2-dimensional parameter spaces (*α*_1_, *β*_1_) or (*α*_2_, *β*_2_).

Step 2: the likelihood of each of the 100 particles are calculated by following Equation 6.

Step 3: the velocity vector of the particle, serving as the guide to search for the optimal solution, was calculated using Equation 7.

(7) $$V_{k}^{'}=c_{0}V_{k}+c_{1}(P_{global}-P_{k})+c_{2}(P_{k-local}-P_{k})$$

where **P**_*global *_is the global optimal solution achieved so far; **P**_*k*-*local *_is the local optimal solution achieved by particle *k*; and *C*_0_, *C*_1_, *C*_2 _are adjustable weight factors used to control searching speed.

Step 4: in the solution space, all the particles are re-positioned based on their current positions and movement velocities calculated in Equation 8.

(8) $$P_{k}^{'}=P_{k}+V_{k}^{'}$$

Steps 1–4 will be iterated until further particle movement cannot result in higher likelihood (defined in Equation 6).

At the convergence, *Z*_*i *_can be interpreted as the probability of differential Pol II binding between two conditions. Although the model derivation is based on ChIP-seq data from MCF7 cells before and after treatment with vehicle or E2 for 3 hours, it can also be equally applied to OHT-MCF7 (+/- E2 treatment). In practice, the solution of the E-M algorithm converges in only 5 to 6 cycles.

## Results and discussion

### Genome-wide identification of Pol II binding in breast cancer cell lines

We tested our model on the Pol II binding quantity in MCF7 and OHT-MCF7 breast cancer cell lines (+/- E2 treatment) derived from the use of ChIP-seq technology. Among all the DNA fragments detected in each sample, we selected only those with high sequencing and matching quality that could be mapped to unique genomic locus. This pre-filtering step sufficiently removed background detection noise, and 2.59, 2.52, 3.00, and 1.33 million reads passed the above filter in MCF7 control, MCF7 E2-treated, OHT-MCF7 control, and OHT-MCF7 E2-treated samples, respectively. In order to compare Pol II binding quantity within a specific genomic region across multiple samples, the number of detected Pol II fragments was normalized using the total number of matched fragments in each sample, based on the assumption that the total number of DNA-binding Pol II would be similar in different cell types under different biological conditions.

### Estrogen-induced changes in Pol II binding quantity in two cell types

**Figure 2:**
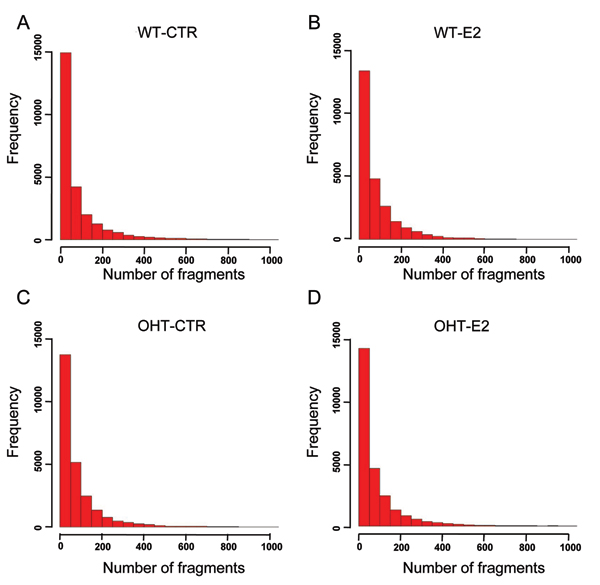
Histogram of the number of Pol II binding fragments found within over 20,000 open reading frames in four samples including (A) wild type MCF7 control, (B) wild type MCF7 treated with E2, (C) OHT-resistant MCF7 control, and (D) OHT-resistant MCF7 treated with E2 samples.

As the Pol II binding quantity in each gene transcript region reflects the expression level of the corresponding gene, we analyzed whole genome Pol II binding distributions for the four breast cancer cell samples. For MCF7, E2 treatment resulted in a slightly higher Pol II quantity distribution (Fig. 2, upper panels). Global gene profiles tended to be higher after E2 treatment, as reflected by a decrease in the number of genes showing a lower level of expression and an increase in the more highly expressed genes (Fig. 2). In control MCF7 samples, ~15,000 genes contained less than 50 ChIP-seq-derived DNA fragments in the gene transcript region (Fig. 2A), decreasing to ~13,000 after E2 treatment (Fig. 2B), a trend not observed in OHT MCF7 cells.

This observation is consistent with the nature of the MCF7 cell line and the OHT-MCF7 subline, representing hormone-dependent and -independent breast cancer, respectively, and was also seen in the mean values and standard deviations for these samples (Table 1). After normalization, all four MCF7 cell lines had the same mean value for Pol II quantity; however, the slight decrease in standard deviation for Pol II quantity after E2 treatment of wild type cells indicates that a greater number of genes were expressed at a higher level. Furthermore, in the OHT cells, which are less sensitive to E2 stimulation compared to the parental MCF7 cell line [13], the standard deviation of Pol II quantity distribution remained essentially unchanged (Table 1).

**Table 1:** The mean value and standard deviation of Pol II quantity in gene transcript regions in MCF7 and OHT-resistant MCF7 cells before and after E2 treatment.

	Wild Type		OHT-resistant	
	Control	Treatment	Control	Treatment

Mean Value	90.2	90.2	90.2	90.2
Standard Deviation	202.8	162.8	172.7	171.9

### Genome Pol II quantity changing level analysis

**Figure 3:**
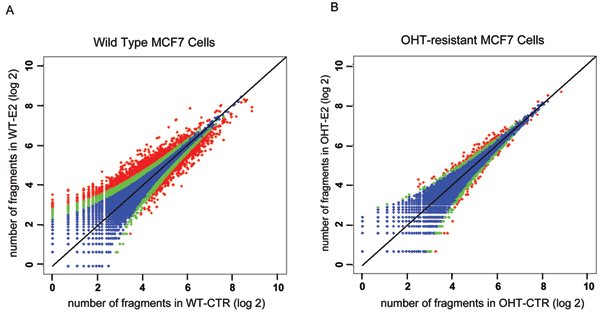
Scatter plot of Pol II binding quantity in control and E2 treated samples: (A) wild type MCF7 cells and (B) OHT-resistant MCF7 cells. Blue, green, and red dots indicate genes with no change (Z < 0.1), minor change (0.1 ≤ Z < 0.9), and major change (Z ≥ 0.9), respectively.

Because no replicates were available for the test data, a *Poisson *mixture model was used to identify estrogen-induced differences in Pol II binding quantity in the two cell lines. The results are shown as a scatter plot of the (log2) number of fragments in control and E2-treated samples (Fig. 3; each dot in the figure denotes a gene). This figure demonstrates a clear trend that with the increase of the number of Pol II binding quantity in the gene transcript region, a less relative change is required for a gene to be considered as major change (red dots, *Z*_*i *_≥ 0.9), or minor change (green dots, 0.1 ≤ *Z*_*i *_< 0.9), where *Z*_*i *_is a posterior probability that the Pol II binding quantity changed after E2 treatment. This result demonstrates a critical feature of the *Poisson *mixture model: more weight is given to high abundant signals, while additional penalties are imposed on genes with low abundant quantities. The motivation is that additional relative changes are required to separate low abundant signals from background noise, because high signals are less sensitive to background noise. Consistent with previous observations [13], wild-type MCF7 cells have more gene targets with an altered quantity of Pol II than OHT MCF7 cells.

**Figure 4:**
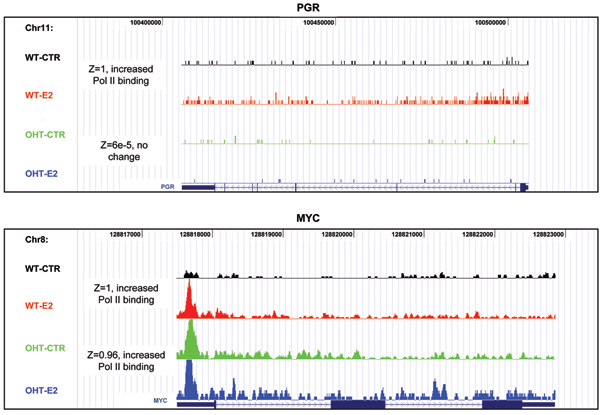
Examples of Pol II binding quantity in the open reading frame of (A) PGR, progesterone receptor and (B) MYC in all four samples.

**Figure 5:**
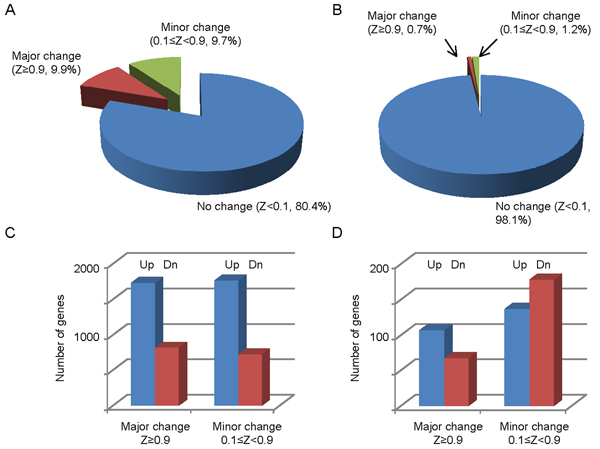
Changes of Pol II binding quantity in wild type MCF7 cells and OHT-resistant MCF7 cells before and after E2 treatment. Percentage of genes with no change (Z < 0.1), minor change (0.1 ≤ Z < 0.9), and major change (Z ≥ 0.9) in (A) wild type MCF7 cells and (B) OHT-resistant MCF7 cells. Number of genes with increased and decreased Pol II binding quantity in (C) wild type MCF7 cells and (D) OHT-resistant MCF7 cells. Dn = Down.

Fragment distribution in the gene transcript region of two genes falling in the "major change" catergories, PgR (progesterone receptor) and MYC, two well known ER*α *targets in hormone-dependent breast cancer [14,15] are shown in Fig. 4. Pol II binding quantity in the gene transcript region of PgR was significantly increased by E2 in wild-type MCF7, but no change was detected in OHT-MCF7 (Fig. 4B). Pol II binding in MYC, however, was significantly increased in both MCF7 and OHT-MCF7 cells. Overall, in wild type MCF7 cells, Pol II binding quantities in the gene transcript region of 9.9% and 9.7% of the genes were classified as major (Z ≥ 0.9) and minor (0.1 ≤ Z < 0.9) changes, respectively (Fig. 5A). These percentages, however, dropped almost 10 fold to 0.7% and 1.2% in OHT-resistant MCF7 cells, while 98.1% of genes had the posterior probability of less than 0.1 (Fig. 5B). Furthermore, in wild type MCF7 cells, among the 2,527 and 2,464 genes showing major and minor changes, 68.1% (1,721) and 71.2% (1,754) of the genes demonstrated increased Pol II binding, respectively. In contrast, an increase in Pol II binding was observed in only 61.6% (106) of the major changed genes in the OHT-MCF7 cells. Strikingly, this number decreased to 43.5% (136) in the minor change group, while 56.5% (177) genes contain decreased Pol II quantity in OHT-MCF7 (Table 2).

**Table 2:** The gene numbers and percentage of whole genome in wild type and OHT-resistant MCF7 cells with minor change (0.1 ≤ Z < 0.9) or major change (Z ≥ 0.9)Pol II quantity.

	Wilde type MCF7 Cell Genes				OHT-resistant MCF7 cell Genes			
	
	Up-regulate		Dn-regulate		Up-regulate		Dn-regulate	
	
	Number	Percent	Number	Percent	Number	Percent	Number	Percent
Minor change (0.1 ≤ Z < 0.9)	1754	6.9%	710	2.8%	136	0.5%	177	0.7%
Major change (Z ≥ 0.9)	1721	6.7%	806	3.2%	106	0.4%	66	0.3%
Sum	3475	13.6%	1516	6.0%	172	0.9%	243	1.0%

## Conclusion

We report a *Poisson *mixture model to identify estrogen-induced changes in Pol II binding quantity in wild type MCF7 cells and OHT-resistant MCF7 cells. Despite having only one replicate available, our model successfully identified genes with different Pol II binding quantities from data derived using ChIP-seq technology. This model can distinguish differentially expressed Pol II activities from unchanged Pol II activities using a posterior probability calculated through an empirical *Bayes *approach. The empirical *Bayes *approach utilizes a combination of E-M and PSO algorithms for estimation and optimization in detection of the differential Pol II binding in two biological conditions. In this model, small signals require large changes in binding quantity to reach the same level of significance. The proposed model is unique in its ability to handle the ChIP-seq data without replicates and thus is an excellent tool for laboratories to evaluate preliminary ChIP-seq results. However, it is important to point out that despite the fact that no replicates are required to calculate changing probability, the use of biological replicates to capture persistent measurements in response to certain treatments are strongly encouraged.

## Competing interests

The authors declare that they have no competing interests.

## Authors' contributions

YL and LL designed the study. WF, YL and LL designed and performed the computational modeling and drafted the manuscript. JW, THH, and KPN performed ChIP-seq experiment. All the authors read and approved the final manuscript.
